# A Cross‐sectional observational study on YouTube as an educational resource Evaluating the quality and reliability of videos for family caregivers of persons living with Alzheimer disease dementia

**DOI:** 10.1002/alz70863_110890

**Published:** 2025-12-23

**Authors:** Lindt Ongkingco Alba

**Affiliations:** ^1^ University of the East Ramon Magsaysay Memorial Medical Center, Quezon City, Metro Manila, Philippines; Cardinal Santos Medical Center, San Juan, Metro Manila Philippines

## Abstract

**Background:**

Patients are increasingly using YouTube as a source of health‐related information. This study assessed the quality and reliability of videos on Alzheimer's disease dementia (ADD) available on the platform.

**Method:**

In October 2023, YouTube was systematically searched for ADD‐related videos. Two independent physicians reviewed each video, scoring it using modified DISCERN (mDISCERN) for reliability and the Global Quality Scale (GQS) for content quality. Videos were categorized by goal and assessed for quality, accuracy, comprehensiveness, and specific content.

**Result:**

There were 58 videos included in the study. Using the GQS, 16 videos (28%) were assessed as high quality, 32 videos (55%) as medium quality, and 10 videos (17%) as low quality. Using the mDISCERN scale, 48 videos (83%) were deemed reliable, while 10 videos (17%) were classified as unreliable. Videos from academic institutions and physicians exhibited higher mDISCERN and GQS scores compared to other groups (*p* =  0.004, *p* =  0.005, respectively), and a significant correlation was seen between mDISCERN and GQS (*p* < 0.001).

**Conclusion:**

The majority of YouTube videos on ADD are good to fair quality, covering disease properties, treatment choices, and patient experiences. However, video popularity does not significantly correlate with content reliability and quality. Videos provided by academic institutions and healthcare professionals can help the general population to understand ADD.

